# Food quality influences behavioural flexibility and cognition in wild house mice

**DOI:** 10.1038/s41598-024-66792-6

**Published:** 2024-07-12

**Authors:** Ekaterina Gorshkova, Stella Kyomen, Markéta Kaucká, Anja Guenther

**Affiliations:** 1 RG Behavioural Ecology of Individual Differences, Max Planck Institute for Evolutionary Biology, 24306 Plön, Germany; 2 Zoology and Functional Morphology of Vertebrates, Christian-Albrechts-Universität zu Kiel, Am Botanischen Garten 1-9, 24118 Kiel, Germany; 3 RG Evolutionary Developmental Dynamics, Max Planck Institute for Evolutionary Biology, 24306 Plön, Germany

**Keywords:** Behavioural ecology, Animal behaviour

## Abstract

Environmental change is frequent. To adjust and survive, animals need behavioural flexibility. Recently, cognitive flexibility has emerged as a driving force for adjusting to environmental change. Understanding how environmental factors, such as food quality, influence behavioural and/or more costly cognitive flexibility. Here, we investigate the effects of high-quality versus standard food as well as the effects of different housing conditions on both types of flexibility. Our results show that mice that experienced a poorer diet under seminatural conditions showed greater behavioural but not cognitive flexibility. For cage-housed mice, the results were less clear. However, mice fed a poorer diet performed better in innovative problem-solving, thus showing enhanced cognitive flexibility, which was not apparent in the reversal learning paradigm. The observed differences were most likely due to differences in motivation to obtain food rewards. Additionally, animals on poorer diet had lower brain volume, usually related to lower cognitive task performance at the between-species level. Thus, our study emphasises the importance of environmental conditions on behavioural flexibility at the within-species level, highlights that different test paradigms may lead to different conclusions, and finally shows that cage housing of wild animals may lead to patterns that do not necessarily reflect natural conditions.

## Introduction

Understanding how animals respond to fast-changing environments is a major challenge for evolutionary biologists. Alongside ecological and life history factors, behavioural flexibility plays a key role in facilitating the exploitation of novel or fast-changing environments by allowing animals to take advantage of new opportunities and respond appropriately to new threats^1–3^. Animals can adjust rapidly due to cognitive processes that support optimal foraging, survival, and invasion success. Cognition can thus be thought of as the driving force behind behavioural flexibility^4,5^. Behavioural, and especially cognitive, flexibility are forms of cognition that allow animals to adjust their behaviour to changes in the environment in a short time with limited experience^6,7^

Behavioural flexibility is a case of phenotypic plasticity responsible for adjusting behaviour according to changing circumstances^8^. It does not necessarily involve costly cognitive functions. Behavioural flexibility is often measured by variance partitioning. Raw phenotypic variances are influenced by the among-individual variances (i.e., by how much the mean phenotypes differ from each other). In addition, the variances contain the within-individual variation if individuals have been measured repeatedly. Within-individual variation reflects measurement error and stochastic fluctuations but also contains an element of behavioural flexibility by showing how much an individual exhibits different trait expressions at several time points^9,10^. Cognitive flexibility, which is assumed to be inherently costly, is an integral component of behavioural flexibility and can be described as differences in the ability to learn and remember^5^. Cognitive flexibility is often measured via reversal learning (RL) or in the context of innovation^11–14^ and innovative problem-solving^13^. Innovation is often measured experimentally^14–16^, and there is support for the idea that cognitive abilities underlie innovative problem-solving^15^.

The size of the brain, relative to the size of the body, limits the capacity of animals to process and store information and thus is assumed to affect the ability of individuals to produce flexible responses to environmental challenges^14,17^. In birds and primates, the innovation rate is positively linked with the relative size of the brain^14,18^. Therefore, several studies have used brain size/volume relative to body size as a structural measurement of behavioural and cognitive flexibilities^19,20^.

Cognitive abilities vary considerably both between and within species^21–27^. For example, bird species with larger brains are known to be more flexible and possess enhanced cognitive abilities, as shown by higher rates of innovation^11,20,28,29^. At the species level, woodpecker finches (*Camarhynchus pallidus*) outperform closely related tree finches (*C. parvulus*) in an extractive foraging task^30^. Nevertheless, they showed no advantage in learning speed for a colour discrimination task or reversing their choice of colour cue^30^. At the within-species level, field mice, *Apodemus agrarius* differed in their cognitive abilities depending on whether they originated from rural or urban environments^31^. The present study on field mice indicates that specific environmental conditions may influence cognitive flexibility within species. It is important to note that studies investigating the effects of the environment on cognition at the within-species level in natural or wild settings are relatively scarce. Nevertheless, researchers have recently begun focusing on this subject area^32–35^. This scarcity of research in natural environments may be the reason for the partially contrasting hypotheses in the literature regarding the relationship between the environment and cognition.

The cognitive buffer hypothesis (CBH) suggests that increased brain size leads to increased cognitive and behavioural flexibility for individuals to cope with environmental variability^36^. Studies on climbing perch, *Tropidurus* lizards, woodpecker finches, and blue-tongued skinks have indicated that brain size is linked to learning speed and flexibility to reverse a previously learned association^23,27,37,38^. Therefore, many studies assume a direct link between large brain sizes in relation to body mass and enhanced cognitive abilities^28^. However, there is contradictory evidence regarding this hypothesis in birds and fish^5,24,39^. An explanation for this could be the expensive brain hypothesis (EBH)^36,40^, an alternative hypothesis stating that animals living in harsh conditions may not have enough energy to maintain a large brain. For example, larger brains assist birds in responding to novel conditions by enhancing their propensity to innovate^28^.

Two more hypotheses may be important for interpreting patterns in problem-solving (PS). According to the necessity drives innovation (NDI) hypothesis, which represents an extension of the CBH, the NDI focuses explicitly on innovative problem-solving^41^. One of the presumed reasons that individuals in poor conditions, such as harsh environments, may be more motivated to innovate in resource acquisition is the direct link between competitiveness and physiological condition^41^. However, evidence for this predicted link is scarce^41–46^. In some species, dominant individuals, such as those with access to more resources, typically initiate interactions with objects and exhibit an even greater inclination towards innovation when tested alongside nondominant individuals^42,47^. On the other hand, the free time hypothesis (FTH)^48^ suggests that the availability of free time or energy can stimulate innovation^48^. Additionally, Van Schaik et al.^49^, in their study on orangutans, showed that a lack of predators allows for long, undisturbed periods of independent exploration, which can lead to innovations. There is also evidence of a link between exploration and PS, as demonstrated in a study conducted by Cauchard et al.^50^. They observed that great tits that successfully eliminated an obstacle obstructing their nest exhibited notably faster object contact after landing on their nest box, in contrast to birds that failed to remove the obstacle.

All existing hypotheses predominantly propose the selection of cognitive traits as a driving force, which operates through the relatively slow process of gene-frequency changes. Genetic drift can present a challenge when adapting to rapidly changing environments, as it may not align with the need for quick adjustments. In such cases, phenotypic plasticity can play a crucial role. Phenotypic plasticity broadly explains how the same genotype can exhibit different phenotypes under different environmental conditions^51,52^. For example, a study on *Apodemus agrarius* from rural and urban environments indicated differences in cognition due to plasticity^31^.

Experimental comparisons of behavioural and especially cognitive flexibility within species and across several generations are rare. In our study, we used descendants of wild-caught *Mus musculus domesticus* living in seminatural environments for six generations (F6) and receiving one of two different diets (two enclosures each). Two diets, standard-quality (SQ) and high-quality (HQ), were used in this study. The HQ diet had greater caloric, protein, and fat contents than did the SQ diet. We considered the SQ food to be more challenging because these animals have lower growth and fecundity rates than HQ animals^53^. We showed in previous studies that our mice fed an HQ diet demonstrated a transition towards a less proactive stress-coping style and became risk averse within three generations^53^. We also noted that the raw, phenotypic variance of risk-taking behaviours started to differ between treatments after approximately 2–3 generations, at which time the HQ animals seemed more variable. Thus, this finding suggests that behavioural flexibility, and therefore cognitive flexibility, differs among our food treatments. Here, we formally investigated, across different test scenarios and several generations, whether behavioural and/or cognitive flexibility differs in mice experiencing different food qualities.

In the current study, we systematically analysed the different variance components of risk-taking and novelty-seeking behaviours across generations F3 to F6, both in seminatural conditions and in cage housing (for the last two generations), to test for potential differences in non-costly behavioural flexibility. We hypothesised that animals on an HQ diet would display greater within-individual variability than mice on an SQ diet, based on recent findings from our seminatural populations indicating a greater responsiveness to environmental change in HQ mice (Lopez-Hervas et al., in press). Our hypothesis aligns with the FTH, which suggests that improved dietary conditions may lead to increased flexibility.

Next, we examined cognitive flexibility for the F3 generation (seminatural enclosures) and across the F5 and F6 generations (in cages). In addition, we measured the relative size and volume of the brains of mice fed different diets of F3 animals from seminatural enclosures. Based on the CBH and its extension, the NDI hypothesis, we predicted that in more challenging environmental conditions, such as the SQ conditions, there will be an increase in relative brain size and improvements in cognitive flexibility. This would result in the animals receiving the SQ food performing better than those receiving the HQ food.

## Results

### Behavioural flexibility measured as variance components of mixed-effect models

#### Treatment variances in open field measurements across generations and housing conditions

**Figure 1:**
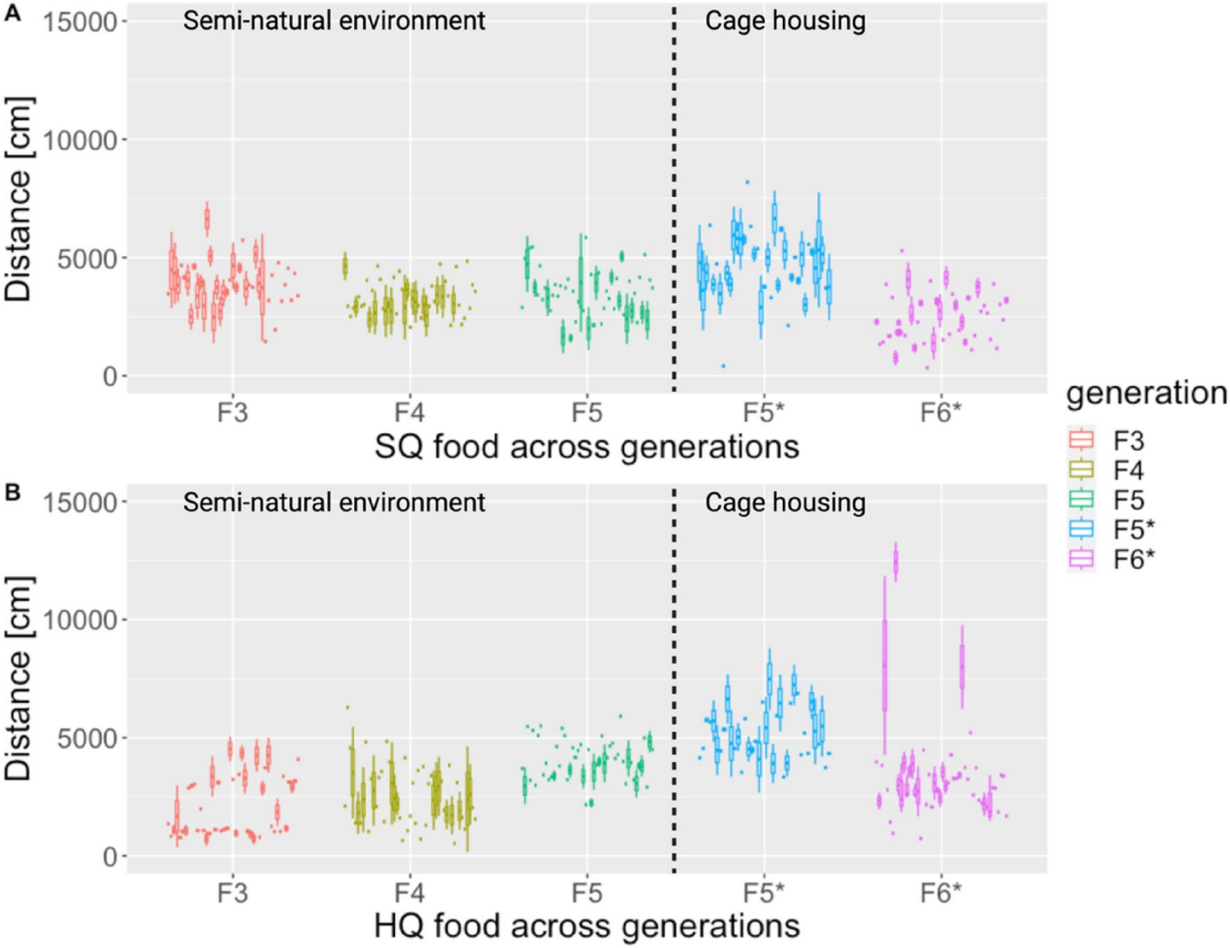
Covered distance in the open field (OF) across generations (F3 to F6) based on food treatment, where each boxplot represents two measurements per mouse with an intertest interval of three weeks: (**A**) SQ mice and (**B**) HQ mice. * indicates that animals were housed in cages, while all others refer to animals housed and tested under seminatural conditions.

We examined potential treatment differences in the behavioural flexibility (indicated by the residual variance of mixed effects models) of risk-taking behaviour measured as the distance covered in Open Field (OF) tests (Fig. 1). Animals from generations F3 to F5 were caught directly from seminatural enclosures, while other F5* and F6* generation animals were housed in cages. All animals were tested twice with an intertest interval of three weeks. Our findings indicate a greater degree of residual variance, and therefore behavioural flexibility, in SQ animals from generations F3 to F5 under seminatural conditions (indicated by more considerable within-individual variance (WIV) components, Table 1). However, this pattern was not observed in the F5* animals from cages, and no difference was observed between treatments, whereas in F6*, mice on HQ had a greater WIV than those on SQ.

**Table 1:** Random effect variance estimates obtained from mixed effect models (using sex and trial as fixed and individual identity as a random effect) for distance covered in an Open Field test.

Generation	F3		F4		F5		F5*		F6*	
	Seminatural environment						Cage-housing			
Treatment	SQ (36)	HQ (41)	SQ (50)	HQ (51)	SQ (50)	HQ (50)	SQ (30)	HQ (29)	SQ (45)	HQ (48)
Random effects										
Total V	1094040	1770007	775534	1773726	1109874	759557	1494595	1600954	1249371	4536451
Among-individual V	361629	1497508	0	1220360	261782	174898	288821	338519	1058593	2861633
Within-individual V (i.e., individual flexibility)	**732411**	272499	**775534**	553366	**848092**	584659	1205774	1262435	190778	**1674818**
	WIV 2.7 times larger in SQ than HQ		WIV 1.4 times larger in SQ than HQ		WIV 1.5 times larger in SQ than HQ		~ Same		WIV 8,7 times larger in HQ than SQ	
Nonoverlapping confidence intervals										
Random effect	− 0.63 (− 186.62, 187.72)	− 0.16 (− 362.98, 379.49)	0 (0.00, 0.00)	− 0.41 (− 300.65, 304.76)	− 0.41 (− 146.23, 142.58)	0.07 (− 120.5392, 115.9662)	− 0.46 (− 196.81, 191.86)	− 2.63 (− 219.37, 215.89)	1.11 (− 317.95, 318.78)	− 2.44 (− 493.34, 475.83)

*indicates that animals were housed in cages, while all other animals were maintained and tested under seminatural conditions. The numbers in parentheses indicate the sample sizes. Random effects refer to the results of the mixed-effects model. The highest value for Within-individual Variation in a generation is highlighted in bold. The nonoverlapping confidence intervals refer to random effects, with 95% confidence intervals around the mean.

## Behavioural flexibility in controlled cages: novel object

For the animals housed in cages, we conducted novel object (NO) tests to measure behavioural flexibility without a cognitively demanding aspect. Similar to the OF tests, each individual was tested twice with an intertest interval of three weeks. The SQ and HQ mice did not differ in any of the parameters for mean trait values (mixed effects models: latency to approach: p = 0.767, t value = 0.298, time spent interacting with the object: p = 0.870, t value = − 0.164 and number of interactions: p = 0.231, z value = − 1.199; Supplementary Figure S1). In the cages, we observed that in the F5* generation the SQ mice showed greater residual variance in terms of latency (Table 2). However, we observed no differences between treatments for the time and number of interactions, similar to the results for OFs from the same generation (Table 1).

**Table 2:** Random effect estimates obtained from mixed-effects models using food and trial as fixed and individual identity as a random effect in novel object tests conducted twice with an intertest interval of three weeks (conducted in F5* generation housed in cages).

Treatment	SQ (21)	HQ (18)
Latency to approach (lmer, Gaussian distribution)		
Total V	213154	45390
Among-individual V	0	29649
Within-individual V (i.e., individual flexibility)	**213154**	15741
	WIV 13.5 times larger for SQ animals	
Nonoverlapping confidence intervals		
Random effect	0 (0, 0)	− 0.18 (− 232.82, 566.75)
Time spent with an object (lmer, Gaussian distribution)		
Total V	724.3	874.2
Among-individual V	143.4	174.0
Within-individual V (i.e., individual flexibility)	580.9	**700.2**
	WIV 0.8 times larger for HQ, i.e., similar for SQ and HQ animals	
Nonoverlapping confidence intervals		
Random effect	− 0.03 (− 23.27, 24.63)	0.03 (− 26.10, 27.11)
Number of interactions (glmer, Poisson distribution)		
Total V	0.08535	0.4178
Among-individual V	0.08535	**0.4178**
	WIV 0.2 times larger for HQ, i.e., similar for SQ and HQ animals	
Nonoverlapping confidence intervals		
Random effect	0.01 (0.59, 0.54)	0.01 (− 0.59, 0.54)

The highest value for Within-individual Variation in a generation is highlighted in bold.

## Cognitive flexibility measured across cognitive tasks

### Cognitive flexibility in seminatural environments: solving escape problems

**Figure 2:**
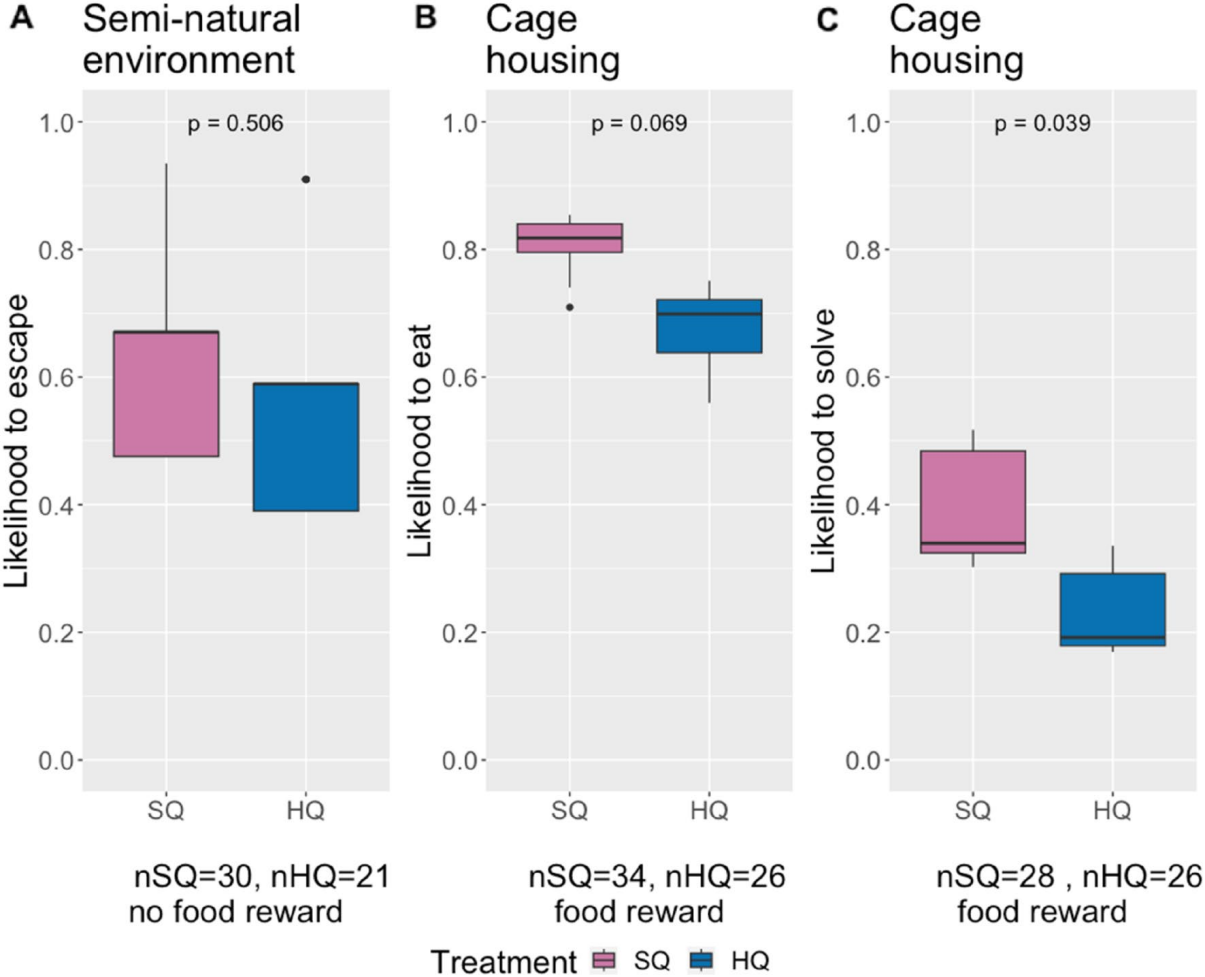
(**A**) Likelihood of escape by problem solving of F3 generation mice from seminatural enclosures averaged across three different escape tasks; (**B**) and (**C**) show results from food-rewarded problem-solving tasks in F5* and F6* mice housed in cages. The results are averaged across three test setups (see Fig. 5) using mixed effects models with food treatment as fixed and individual identity as random effects. (**B**) Likelihood of eating a food reward from the open setup presented one day before the actual problem-solving task. (**C**) Likelihood of solving the food-rewarded problem-solving tasks.

In the F3 generation, we tested the problem-solving performance of the animals in seminatural enclosures using escape paradigms. Averaged across three different escape problems (see Fig. 5), SQ and HQ mice were equally likely to solve the problem of unblocking the route and escaping (p = 0.506, z value = − 0.665) (Fig. 2A). Similarly, we did not observe treatment differences in the means for latency to touch or time spent interacting with the problem during the escape PS tasks, indicating that mice subjected to different food treatments perceived the situation as similarly stressful^54^ and were equally motivated to escape (Supplementary Figure S3).

## Cognitive flexibility in controlled cages: Food-rewarded

### Problem-solving

For the F5* and F6* generations that were housed in a more controlled environment in cages, we conducted three food-rewarded problem-solving tests. Since the motivation to engage in food-rewarded tests might differ for animals that experience different quality food, we added controls by showing the animals open test setups with easy access to the food reward prior to the actual testing. For the latency to approach, time spent interacting, and number of interactions in the food-rewarded PS tasks, mice on both diets showed similar trait average values in both the introduction (open) setup and in the actual test when they needed to solve the closed setup (see supplementary material, Figure S2). However, we observed differences in the motivational (likelihood of eating in the introduction) and cognitive aspects of this task (likelihood of solving in solving) (Fig. 2). While approximately 80–85% of the mice on the SQ diet consumed the treatment from the open set-up, the proportion of HQ mice that consumed the treatment was approximately 65–70% (p = 0.069, z = − 1.820). SQ mice were more likely to solve the task in the closed setup (p = 0.039, z = − 2.068).

## Reversal learning

**Figure 3:**
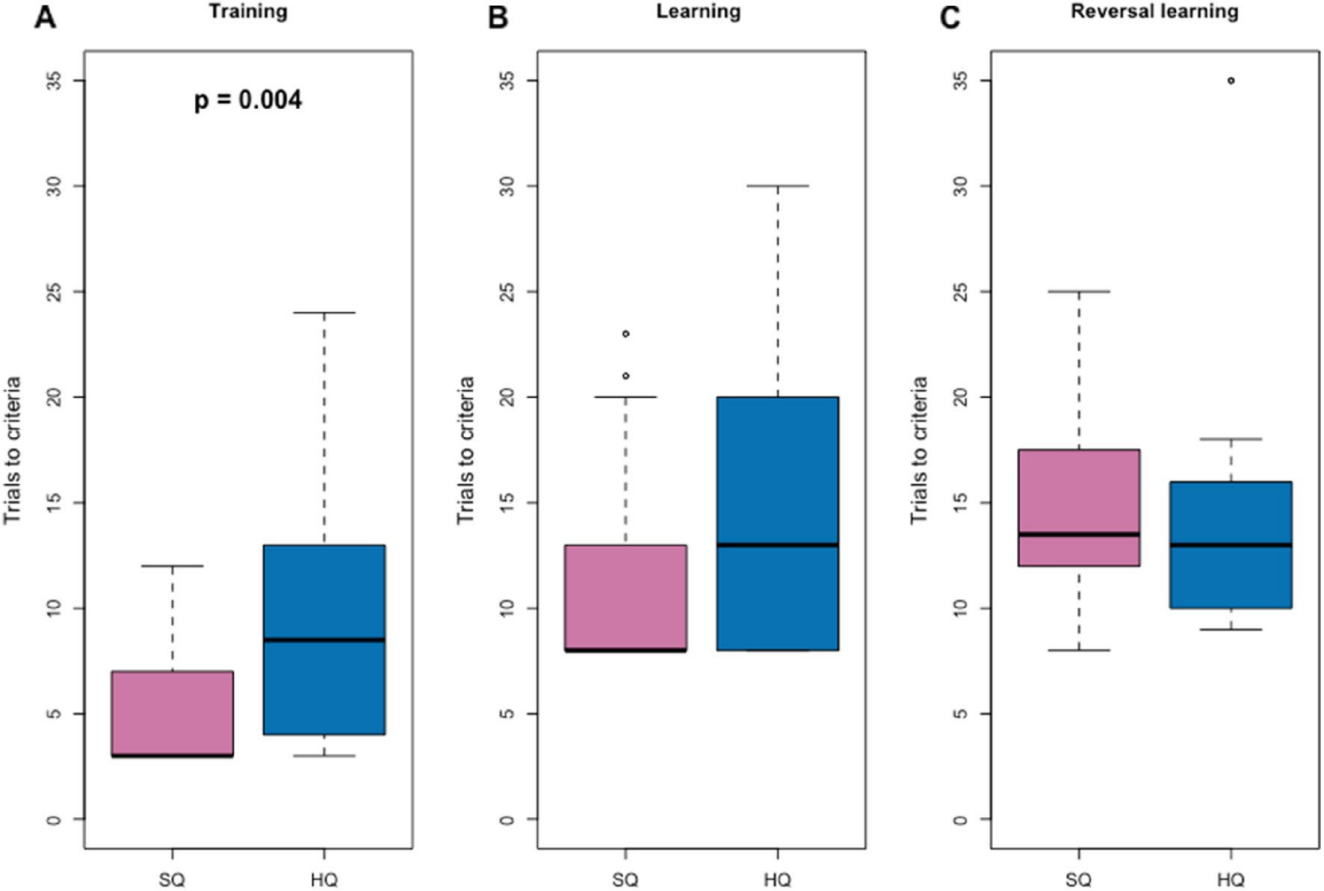
Differences in the number of trials to pass the experimental phase criterion between mice from the F5* and F6* generations fed different diets from cage housing: (**A**) Training phase (RL), N = 42; (**B**) Learning phase (RL), N = 33; (**C**) Reversal learning phase (RL), N = 32.

We used the RL test paradigm as a second setup to explicitly test cognitive flexibility. In the initial training phase, the animals had to learn to find a food reward in a hole on a holeboard (see Fig. 5). Compared with HQ mice, SQ mice reached the criterion of consuming the treat in three consecutive trials more quickly (Fig. 3A, t = 3.01, p = 0.004). In the learning and reversal stages, however, the SQ and HQ mice needed a similar number of trials to reach the passing criterion of receiving eight correct choices in a row (Fig. 3B, C). Additionally, we performed a t test to rule out a colour bias. The results demonstrated that there was no statistically significant difference in colour preference during the learning phase (t = − 0.099, p value = 0.922) or the reversal phase (t = 0.885, p value = 0.383).

## Analysis of brain measurements

**Figure 4:**
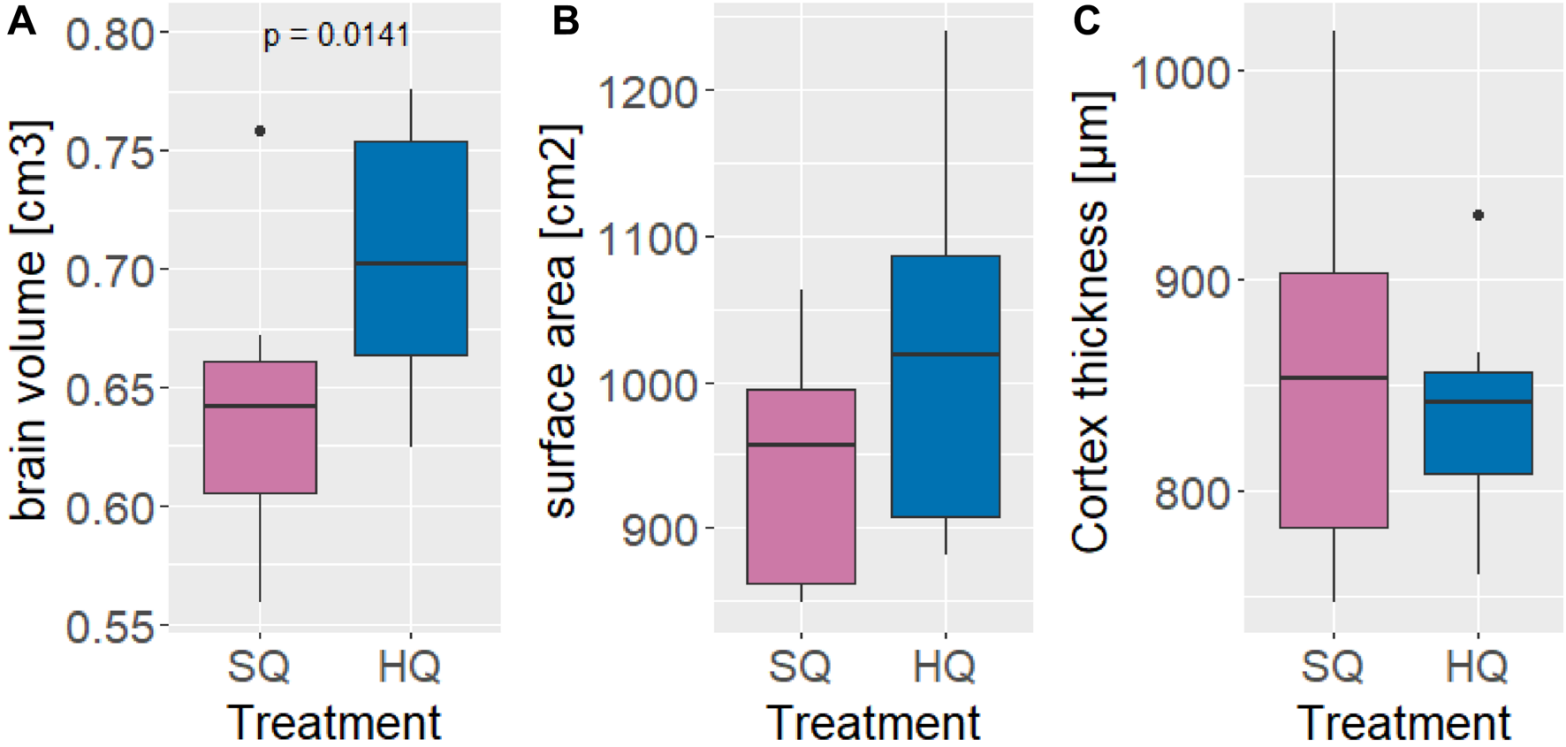
Differences in relative brain volume (**A**), brain surface area (**B**) and cortex thickness (**C**) between SQ and HQ females in the F3 generation.

In generation F3, after the escape PS task was completed, ten females from each food treatment group were sacrificed immediately to analyse the relative brain volume, area and cortex thickness to body size. Brain volume (t = − 2.718, p = 0.014), but not brain surface area (t = − 0.581, p = 0.569) or cortex thickness (t = 0.502, p = 0.622), was greater in HQ animals than in SQ animals (Fig. 4).

## Discussion

Our results show a difference in behavioural flexibility depending on food quality in seminatural environments, but for cage housing, the results are not clear. Mice on the SQ diet from seminatural environments showed greater behavioural flexibility in the open field test. However, mice housed in cages showed no differences between treatments in F5*; however, in F6*, mice on HQ food showed greater behavioural flexibility. In terms of latency in the Novel Object test, mice on the SQ diet housed in cages showed greater behavioural flexibility. However, both treatments had similar effects in terms of time and number of interactions. For cognitive traits, we found differences in motivation as the SQ mice were more likely to eat from open PS setups. While SQ animals performed better in the food-rewarded problem-solving tasks than HQ animals did, there were no differences in the likelihood of escape. Reversal learning showed no differences between treatments except that HQ animals needed more trials in the training phase, again indicating a greater motivation to assess a food reward in SQ animals but indicating similar cognitive flexibility in both treatments. Interestingly, HQ mice exhibited a larger brain volume but comparable brain surface area and cortex thickness to those of SQ mice.

Both behavioural and cognitive flexibility are essential for animal survival. These flexibilities allow animals with limited experience to make quick and appropriate changes in their behaviour in response to external environmental changes^1^. The Open Field test results revealed that mice fed an SQ diet from seminatural enclosures consistently for several generations exhibited greater residual variance, indicating greater within-individual variability in terms of behavioural flexibility. Similarly, SQ mice showed greater behavioural flexibility in the latency to approach an unfamiliar object in their home cage. These results align with the necessity drives innovation hypothesis, which predicts greater flexibility for animals living in harsh environments. Contrary to our results, in female zebra finches, developmental dietary restriction significantly reduced the amount of within-individual variance in the activity rate in the novel environment^55^. Studies in insects have shown that feeding on a low-quality diet (rich in carbohydrates) increases intraindividual stability and repetition of behaviour, therefore reducing behavioural flexibility, again conflicting with our findings^56^. Most other available studies used nutritional restriction rather than changing the quality of the food available and usually only tested for effects on mean-level behaviours rather than considering variances. The mean level is insufficient to fully understand the impact on a particular behaviour, whereas variances can show the diversity in the behaviour of each individual^8,57^, which precludes general conclusions about how animal diet and habitat affect various aspects of behaviour. For mice born and raised in cages, differences regarding behavioural flexibility between treatments disappeared in the F5* generation (Open Field test) or were found only for specific variables (Novel Object test) despite continued food treatment. Interestingly, in F6*, we observed that mice on the HQ diet showed greater within-individual variance than did mice on the SQ diet (Open Field test), which is the opposite of what we observed in a seminatural environment. These findings show that behavioural flexibility is less consistent among cage-housed animals. In a seminatural environment, animals need to allocate their resources to various aspects ranging from territory defence to reproduction, whereas in a cage, conditions are much simpler and less constrained in terms of the need to allocate resources. In addition, we also observed a difference in the active time of our animals, which could also have influenced our results. The duration of activity differed between animals in seminatural conditions and those housed in cages. Animals housed in cages are less synchronised in their activity and are awake longer in the morning; this is not the case for animals in seminatural enclosures. Previous studies also revealed differences in circadian rhythms between rodents in captivity and natural settings^58–60^. Finding such striking differences between animals housed under close-to-natural versus cage environments fuels the ongoing discussion about the validity of behavioural estimates taken in captive laboratory environments^61,62^.

Cognitive processes are known to be relatively energy intensive. Thus, we expected HQ mice to exhibit better cognitive performance than SQ mice, as shown in laboratory studies^63^. Contrary to our expectations, SQ mice demonstrated greater success in solving tasks when offered a food reward (in cages). Interestingly, these differences were not observed when mice needed to solve a problem to escape without a food reward under seminatural conditions. In reversal learning, we also did not observe a difference between the two treatments when a food reward was present. SQ mice outperformed HQ mice only during the training phase, which did not require demanding cognitive activities. Escape-based problem-solving generally showed higher solving probabilities than the nonstressful version that offered a food reward that we used in cages. While the need to escape from an unpredictable situation elicits an immediate stress response in mice, this is not the case when a food-rewarded problem is offered^54^, potentially explaining the differences in solving success. The lack of significant treatment differences for reversal learning may be due to the small sample size, as only those animals that succeeded in learning could move to the reversal learning phase. Alternatively, the observed treatment differences in food-rewarded PS may have been caused by differences in motivation rather than differences in cognitive flexibility. Our investigation highlights distinct differences in motivation between the two treatment groups. Since mice showed no difference in interest in the set-ups, the latency to approach for both groups was the same. However, the mice on the SQ diet showed greater motivation for the treats, as they were more interested in eating the treats from the open set-ups. Based on our results, we propose that the differences in the problem-solving rate could be attributed to the greater interest of mice on lower-quality diets in seeking supplementary nutritional resources.

Motivation is predicted to be crucial in steering innovative behaviour and influencing every phase of the innovation process^41,64^. While motivation is frequently viewed as a factor that might introduce complexity and is sometimes ignored in investigations of problem-solving task mechanisms^16^, motivation also forms the basis for the necessity drives innovation hypothesis^12^. A rarely examined premise is that hunger or lack of nutrients serves as the driving force behind innovative behaviour. As a result, food-related motivation possibly influences the extent to which animals are inclined to exert effort for sustenance, thus potentially impacting their performance in behavioural assessments that use food as a form of positive reinforcement^65^. If, as in our study, a correlation exists between motivation and problem-solving outcomes, this raises the possibility that problem-solving scores alone might not be entirely dependable as indicators of cognition^66^. Some studies, for example, in great tits (Parus major), have observed the effects of motivation on problem-solving success^67^*.* Birds deprived of food one hour before the trial (and therefore more motivated) were more successful at solving problems than birds with lower motivation (i.e., food was accessible at all times)^67^. Thus, our results highlight, on the one hand, that experimental studies should ensure that all animals are equally motivated prior to testing. On the other hand, the impact of motivational differences on task engagement needs to be better understood if we aim to reliably test animals from different environments in the wild.

While our behavioural observations mainly supported the cognitive buffer and necessity drives innovation hypothesis when we found differences in behavioural or cognitive flexibility, the analysis of relative brain measurements contradicted this hypothesis. Mice on the HQ diet had greater relative brain volume, suggesting that animals on richer nutritional diets indeed had more energy to maintain larger brains, which aligns well with the expensive brain hypothesis. Moreover, we have not found evidence that a larger brain corresponds to better cognitive ability. Recently, a few studies have argued that whole-brain size/volume may not be a reliable marker of cognitive complexity since different brain regions possess specific functions that can evolve separately and respond differently to various selection pressures^68,69^. Nevertheless, in both avian and primate taxa, including families in birds and species in primates, higher levels of innovation generally increase with relative full brain volume^70–75^. Similarly, different studies investigating how different environments affect brain size/volume generally support the idea of the expensive brain hypothesis^36,40,76^. Likewise, our results also confirmed that more available energy translates into larger brains. Nevertheless, the link between a larger brain and cognitive ability in our mice was not apparent, at least in tasks commonly used to assess cognitive flexibility.

## Conclusion and outlook

Primarily, our results revealed an increase in relative brain volume among animals exposed to a high-quality diet for multiple generations. However, behavioural and, in some contexts, cognitive flexibility were greater in animals with smaller brains subjected to lower food quality. We hypothesise that differences in motivation to access a food reward may be the driving force behind differences in problem-solving performance. Understanding how environmental effects drive and shape behavioural and cognitive flexibility is important because animals need these traits to succeed in human-altered environments. A study testing striped field mice *(Apodemus agrarius)* originating from urban versus rural habitats revealed that urban animals showed higher levels of reversible behavioural plasticity^77^ as well as higher levels of problem-solving success^31^. In contrast, common voles (*Microtus arvalis)* displayed increased boldness and exploratory behaviour within their natural surroundings, as well as greater behavioural flexibility, than did voles in urban areas^78^. Acknowledging observed differences in behavioural flexibility raises questions concerning the costs animals might have to pay for elevated levels of flexibility and the mechanisms underlying such heightened flexibility. The different mechanisms, such as nonrandom sorting, microevolutionary changes within populations or phenotypic plasticity, that were observed in our study may drive differences in flexibility across different timescales.

## Methods

### Study subjects and housing

In our study, we used *Mus musculus domesticus* housed under seminatural conditions (Part I of our study) or transferred to cage housing (part II). The descendants of the mice originated from the Cologne/Bonn region in Germany and have been maintained for > 10 years in our laboratory. To start this series of experiments, a total of 80 males and 80 females from sixteen unrelated breeding pairs were equally distributed across four seminatural enclosures. Generation numbers (F1-F6) in this study started with the introduction of mice to seminatural environments. Seminatural enclosures (N = 4; size = 19.6 m^2^) were designed to resemble the natural environment of house mice, i.e., a barn or another human shelter^79^. The enclosures contained thirteen nest boxes equipped with wood chips, different nesting materials, and nine food and water stations (for details, see^53^). The temperature and photoperiod followed natural seasonal variations, but we prevented temperatures from falling below 10 °C in winter. Once a month, all individuals of a population were captured and weighed, and new animals received a subcutaneous individual RFID chip for recognition. To prevent social crowding, we removed old animals, i.e.*,* animals of the older generation, from the enclosure as soon as the population size increased above 140 animals, the capacity of our enclosure.

Observations from natural house mouse populations showed that our seminatural enclosures produce similar patterns of population density, population fluctuations, and, most importantly, average relatedness and inbreeding between individuals^79,80^.

Mice were fed either SQ (Altromin 1324, N = 2 enclosures) or HQ diet (Altromin 1414, N = 2 enclosures). The SQ diet contained 3227 kcal/kg of metabolisable energy (24% from protein, 11% from fat, and 65% from carbohydrate), and the HQ diet contained 3,680 kcal/kg (28% from protein, 22% from fat, and 50% from carbohydrate). All animals used in the behavioural tests are listed in Supplementary Table S2.

Animals in part II of the study were maintained in a system of two type III Perspex cages (38 × 22 × 15 cm) connected by a tube, with each pair of cages provided with bedding, food according to their diet, water, two houses and a running wheel. To obtain animals of the F5* generation to be transferred to cage housing, we caught young animals of approximately one month of age during the monthly population monitoring. We removed two or four young animals (depending on the family size) from each family and transferred them to cage housing. A family usually consists of one adult male, several females and their dependent offspring. Thus, young animals knew each other and might have been (half)-siblings. After completion of the experiments, we formed breeding pairs using each female originally from one of the seminatural enclosures and a male originally from the other seminatural enclosure that had the same food type. Each cage system contained two mice from a same-sex sibling pair. Animals were maintained under natural light conditions with additional artificial light from 8 am to 4 pm at a temperature of 18–23 °C. All experiments with cage-housed mice were conducted from 8 to 11 am. The tested animals were separated directly before each experiment by gently directing one animal into one of the cages and inserting a wire separation in the connecting tube.

## Treatment variances in open field measurements across generations and housing conditions

To capture mice from seminatural environments, twenty live traps were placed into an enclosure for 4–5 h, depending on sunset timing, during evening twilight hours. The traps were checked every 10–15 min, and the mice that were not used for the experiments were released immediately. Mice tested in the OF were transferred to the testing room in the trap and directly released into the OF. To test mice from the cages, cages with mice were transferred to the room with the OF. To minimise animal stress, we tested only one mouse from each cage per day. The open field test consisted of a 60 × 60 cm^2^ white box with 70 cm high walls to prevent the mice from jumping out. This test was developed to test risk-taking behaviour since rodents tend to avoid open areas and exhibit thigmotaxis^81^. For five minutes, we recorded the time spent in the central zone (the inner 30 cm square) of the OF and the distance moved during this time using automatic tracking software (TSE Systems GmbH, Germany). In both studies, the animals were retested after approximately three to four weeks. We used the two measurements of the OF test to test for differences in behavioural flexibility (indicated by the residual variance of mixed effects models) of noncognitively challenging behaviours.

## Behavioural flexibility in cages: novel object

For the animals living in cages (i.e., the F5*/F6* generation), we conducted a novel object test to assess curiosity as a potential second, noncognitively challenging personality trait of the mice. First, we aimed to test whether potential differences in response to novelty between food treatments affected PS or learning. Furthermore, we aimed to test for differences in overall behavioural (but noncognitive) flexibility between treatments, comparable to the behavioural flexibility tested in the OF test, while animals lived in seminatural enclosures. For this purpose, we used an object (4 × 4 × 4 cm) made from LEGO, which was made of different colourful bricks and shapes, and placed it in a cage after separating the pairs. The interaction of the mouse with this object was then video recorded for one hour, during which we measured the latency until the mouse first touched the object, the number of interactions and the time spent with the object. A second test for the novel object was carried out three weeks later to assess among- and within-individual variance components. In total, we had four slightly different objects, and each mouse received a random new object during the first and second trials.

## Cognitive flexibility in a seminatural environment: solving escape problems

**Figure 5:**
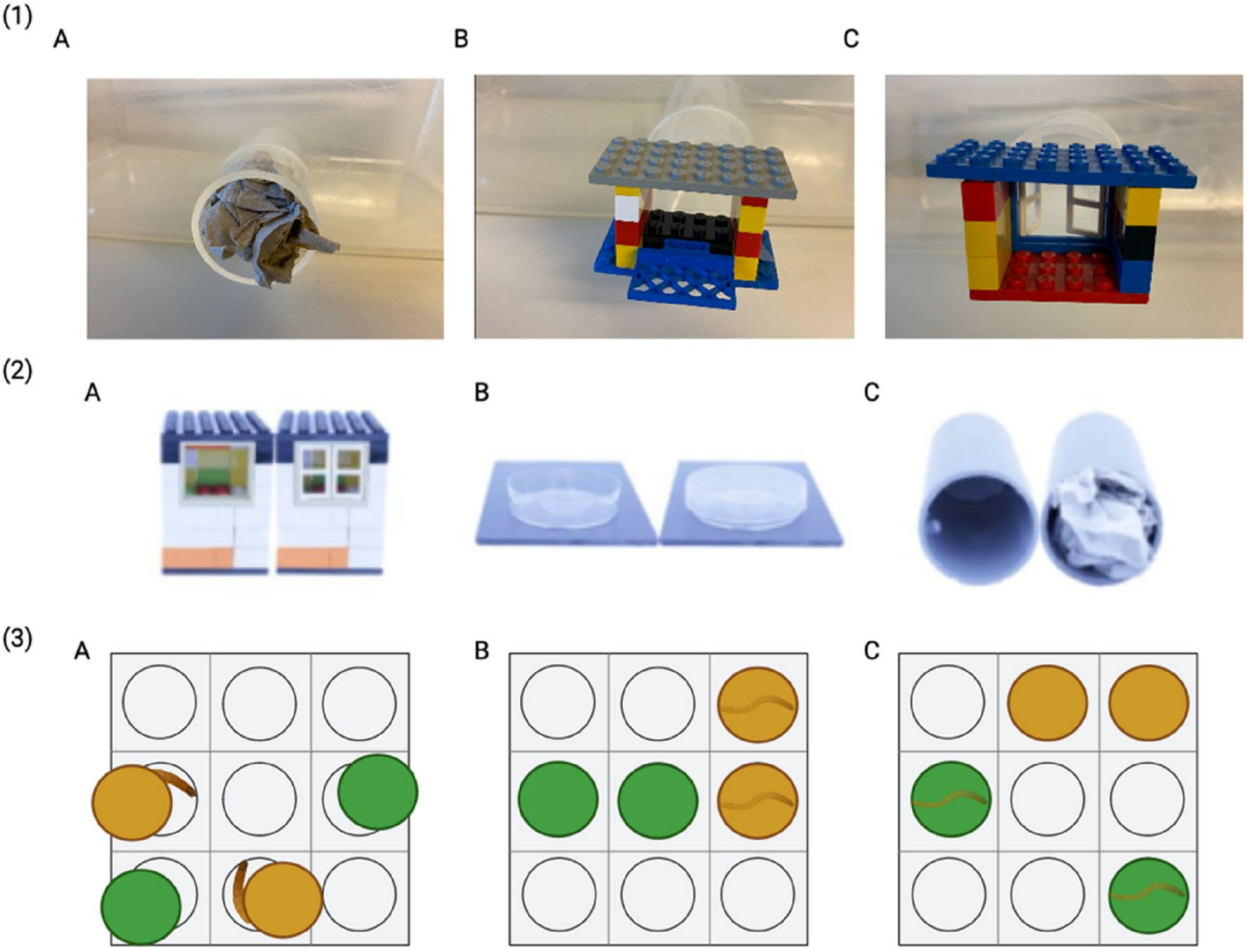
Test set-ups: (1) Escape problem-solving set-ups: (**A**) Exit blocked by paper (mice need to pull or push out paper horizontally), (**B**) Exit blocked by lever (mice need to push the lever down), (**C**) Exit blocked by LEGO window (mice need to pull the window to the sides); (2) Food rewarded problem-solving set-ups: (**A**) LEGO house: open and closed, (**B**) Petri dish: open and closed, (**C**) tube: open and closed; (3) Reversal learning set-ups: (**A**) Training phase, a certain colour (either yellow or green) is assigned to each mouse, the mealworm is not covered, and the mouse has easy access to it; (**B**) Learning phase, this time the mealworm is covered with a lid of the same colour as in the Training phase, the mouse must lift the lid to get the worm; (**C**) Reversal learning phase, the colour behind the mealworm changes, the mouse must lift the lid to get the worm.

We used three escape tests to test the PS abilities of mice fed different diets directly under seminatural conditions. The problems used here were the same as those used previously in a study in house mice^82^. The problems were chosen to be similar in terms of difficulty to solve but to rely on different solving actions. The problem-solving actions that needed to be performed were similar to those required to access food rewards in the PS task using food motivation. Mice were caught using a similar live-trapping procedure to that used for the OF tests. A mouse was introduced to one of the escape cages, and the cage was directly placed in the seminatural enclosure close to the capture point (Fig. 5 (1)). The escape cages contained no shelter or other objects to facilitate interaction with the problem. The mouse was given fifteen minutes to escape from the cage, and if it did not manage to escape it was released. The latency to start interacting with the problem, the escape latency, and the escape success (yes or no) were video recorded under red light.

## Cognitive flexibility in controlled cages: Food reward problem-solving

### Habituation

To prepare animals to engage willingly in further cognitive tests (PS and RL), a mealworm or a sunflower seed was placed on a plastic plate for one hour after separating pairs. After an animal had shown a clear preference for either the worm or the seed, we proceeded with the preferred item for each mouse. It was then checked whether the mouse ate the mealworm or seed within the given time. Animals that ate the food reward for three consecutive attempts, i.e., on three consecutive days, advanced to further tests.

## Problem-solving

The animals were given three PS tasks, each with a mealworm or sunflower seed as a food reward, depending on individual preference. These problems included a LEGO house, a Petri dish, and a tube (Fig. 5(2)). To open the first set-up, the animal had to open the window at the LEGO house (Fig. 5, (2) A); in the second set-up, the mice had to lift the lid of the Petri dish (Fig. 5, (2) B); and in the third condition, the mice had to remove the paper from the tube (Fig. 5, (2) C). Animals were given one trial per day. Each setup was first presented openly (the animal had free access to the food reward), and on the day after the mouse was presented with the closed one. Both sessions were video-recorded for one hour. We measured the latency to approach the open or closed set-up, the results (whether the reward was eaten from the open setup or not, and whether the problem was solved or not), and both the number of interactions and the time mice spent interacting with the set-up.

## Reversal learning test paradigm

### Training phase

For the RL, we used a setup with nine pits and four lids in two different colours (green and yellow), colours that mice are known to be able to differentiate^83,84^ (Fig. 5(3)). In the training phase, the mice got to know the set-up. We placed mealworms or sunflower seeds in 2 pits without closing them. The lids were placed next to the pits, and then this setup was placed in the cage. After one hour, we checked whether the food reward was eaten. When the mouse ate the reward three consecutive times, it participated in the ‘learning’. If the mouse did not pass the required criterion in 35 attempts, we considered that the mouse was not able to pass this phase and it did not participate in further phases of the reversal learning paradigm.

## Learning phase

All mice were assigned a specific colour under which the food reward was located during the ‘learning’ stage. Each trial lasted one hour, with the reward positions changed between trials to avoid place and side learning. As soon as the mouse opened at least one lid with the correct colour the first eight consecutive times, we considered that the mouse had learned the association. The chance of opening the right colour the first eight times in a row is p = 0.0039 and thus differs significantly from chance. Mice meeting this criterion could be moved to ‘reversal learning’. If the mouse opened the first lid of a different colour, the attempt was considered a failure. As in the training phase, if a mouse did not reach the required criterion in 35 attempts, it was considered not able to pass this phase and did not continue on to the next phase.

## Reversal learning phase

In ‘reversal learning’, the mouse was presented with the same setup, but the colour behind which the food reward was placed changed. Similar to the ‘learning’, each trial lasted for 1 h, with the position of the reward changing between the trials. The phase was considered passed only after eight consecutive openings of the correct lid. We counted the number of trials that met the criterion. In all phases of this experiment, the animals were subjected to one trial per day. If the mouse did not pass the desired criterion in 35 attempts, we considered the mouse to be incapable of passing the phase.

## Brain measurements and analysis

### Sample preparation and tissue contrasting

Adult mice were euthanised immediately after capture from seminatural enclosures by CO_2_ at the age of 8–9 months. The brain was dissected carefully from the skull to preserve its integrity and washed quickly in ice-cold PBS. For tissue contrasting, a previously described protocol was followed^85^. Briefly, the samples were fixed in freshly prepared 4% paraformaldehyde (in PBS) and rolled slowly at + 4 °C for 24 h. The samples were dehydrated in an ethanol gradient (30%, 50%, 70%, 90%) with slow rotation for 24 h at + 4 °C. The samples were transferred to 1% iodine in 90% methanol and contrasted for 24 h with slow rotation at + 4 °C. Once the iodine staining was completed, the samples were rehydrated in a reverse ethanol series (90%, 70%, 50%, 30%) for 24 h at + 4 °C and stored in 30% ethanol until they were mounted for scanning. To avoid moving artefacts during scanning, each sample was embedded in 1% agarose gel (A5304, Sigma‒Aldrich) in a thin-wall polyethylene tube, and the tube was fixed firmly to the holder of the CT scanner using tape.

## Micro-CT and image processing

µCT was performed using a Bruker SkyScan 1276 (Kontich, Belgium). A 90 kV acceleration voltage and 50 µA tube current were applied, the exposure time was set to 572 ms, and 4 images were averaged to reduce scanning noise. The samples were scanned with a voxel resolution of 8.22 µm. Nine hundred projections were captured over the 360° rotation. 2D projection images were transformed into 3D volumes using the reconstruction software NRECON (Bruker Corporation, Kontich, Belgium). Scanning artefacts, such as beam hardening, ring artefacts and misalignment, were corrected during image processing. Reconstructed images (see Supplementary Figure S6 for a representative example) were imported to Avizo 3D Pro Software (Thermo Fischer Scientific, Konrad-Zuse-Zentrum, Berlin, Germany), and the 3D volume of the sample was segmented using the “Magic Wand” tool. The total volume and surface area of the brain were quantified to assess the variation between individuals. Additionally, cortical thickness was measured manually between the anterior ventral temporal cortex and prefrontal cortex.

## Ethics declarations

According to national guidelines, project parts potentially involving harm or stressful situations for animals, but not behavioural observations, must be approved. Accordingly, for animals tested in the OF and problem-solving tests in seminatural enclosures, experiments were approved by the Ministerium für Landwirtschaft, ländliche Räume, Europa und Verbraucherschutz; Referat IX 55 Tierschutz under licences V244 – 12,767/2019 and V244 – 31,223/2019 (62–5/19). Sacrificing animals for brain measurements was approved by the animal welfare officers of Kiel University under permit number 1158. The housing of wild mice is approved and regularly controlled by the Veterinäramt Plön under licence PLÖ-004,697. All animals included in the study were handled, and procedures were carried out according to national and ARRIVE guidelines.

## Statistical analysis

For a complete overview of the sample sizes used per experiment, please see Supplementary Table S2. All model outputs are shown in Supplementary Tables S3–S21. We used the mixed effects model from the lme4 package^86^ to analyse all variables for which we had repeated data per individual for the OF, novel object and PS tasks. We used linear models for the reversal learning paradigm and brain measurements. In the case of mixed effects models, animal identity was always included as a random effect. Model assumptions of heteroscedacity and Gaussian error distribution were checked visually by diagnoses of qq-plots. Several other variables were finally modelled assuming a Poisson distribution (number of interactions with a novel object, number of interactions with PS test setups). Binary variables (solved or not solved) were modelled assuming a binomial distribution.

To test for behavioural flexibility using variance components in an Open Field test, we used the distance covered in the OF test and sex and trial as independent fixed effects. We first fitted a model per generation (F5 modelled separately for animals in cages and seminatural enclosures) to test for treatment differences in mean trait expression. In addition, we fitted two separate models, each per treatment within generations (the results are shown in Table 1). The residual component of the random effects part signifies unexplained variability and contains the behavioural flexibility element within each group. We used a similar approach to investigate three variables: latency to approach, interaction time and number of interactions from the novel object test. Initially, one model, including the food treatment and the trial, was modelled to investigate treatment differences. Then, two models were fitted separately for each treatment (the results are shown in Table 2). Additionally, we used Bayesian methods to analyse our data using the “arm” package. We simulated 5000 values from posterior distributions and extracted random effects to explore variability across groups. Furthermore, we calculated the mean of these random effects and computed 95% confidence intervals around the mean.

To analyse problem-solving performance (both escape and food-rewarded), animal responses across the three test setups were used. The models included diet as a fixed variable and individual identity as a random variable. The likelihood to solve was modelled binomially, while the latency to interact with the setup and the interaction time were modelled assuming a Gaussian distribution.

To analyse the reversal learning task, we fitted a total of three linear models with diet as the main factor for the training, learning and reversal stages. Finally, for the analyses of brain measures, we fitted three more linear models using diet as the main effect for brain volume, brain surface area and cortical thickness. Prior to the analyses, the brain measurements were standardised by the individual’s body mass taken just before euthanasia.

### Supplementary Information

Supplementary Information.

## Data Availability

The data have been uploaded as supplementary material (“Supplementary Material Data”).
