# Supplementary material for: Food quality influences behavioural flexibility and cognition in wild house mice
Source: Sci Rep. 2024 Jul 12;14:16088. doi: 10.1038/s41598-024-66792-6 (PMC11245467; doi:10.1038/s41598-024-66792-6)
Supplement: Supplementary file 1 — Supplementary Information. [file 41598_2024_66792_MOESM1_ESM.docx]

**SUPPLEMENTARY MATERIAL**

**Food quality influences behavioural flexibility and cognition in wild house mice**

Ekaterina Gorshkova^1,2*^; Stella Kyomen^3^, Markéta Kaucká^3^; Anja Guenther^1^

^1^ RG Behavioural Ecology of individual differences, Max Planck Institute for Evolutionary Biology, 24306 Plön, Germany

^2^ Christian-Albrechts-Universität zu Kiel, Zoology and Functional Morphology of Vertebrates, Am Botanischen Garten 1-9, 24118 Kiel, Germany

^3^ RG Evolutionary developmental dynamics, Max Planck Institute for Evolutionary Biology, 24306 Plön, Germany

**Corresponding author**:

**E-mail:** gorshkova@evolbio.mpg.de

Supplementary Figure S1.Compairison of interaction with novel object in SQ and HQ mice: A) difference in latency to approach, B) difference in time of interaction, C) difference in number of interactions.


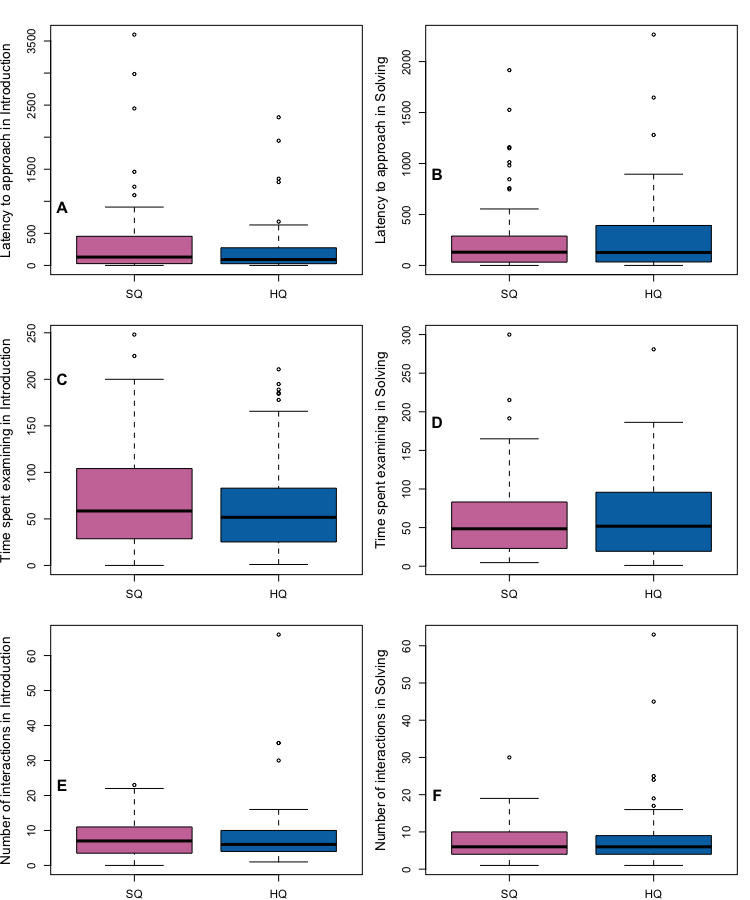


Supplementary Figure S2. Interaction with food rewarded problem solving set-ups in SQ and HQ mice: A) latency to approach in introduction; B) latency to approach in solving; C) time of interaction in introduction; D) time of interaction in solving; E) number of interactions in introduction; F) number of interactions in solving.

Supplementary Figure S3. Latency and time in Escape Problem Solving from semi-natural environment

Supplementary Figure S4. Repeatability between the first and second trials in SQ and HQ mice: A) repeatability in latency, B) repeatability in time of interaction, C) repeatability in numbers of interaction.

Supplementary Table S1– Estimates for effect of food on RL phases

| Phase | Sample size | t value | p-value |
| --- | --- | --- | --- |
| Initial habituation | 47 | -0.705 | 0.484 |
| Training | 43 | 3.055 | **0.004** |
| Learning | 34 | 1.672 | 0.104 |
| Reversal learning | 33 | -0.057 | 0.955 |


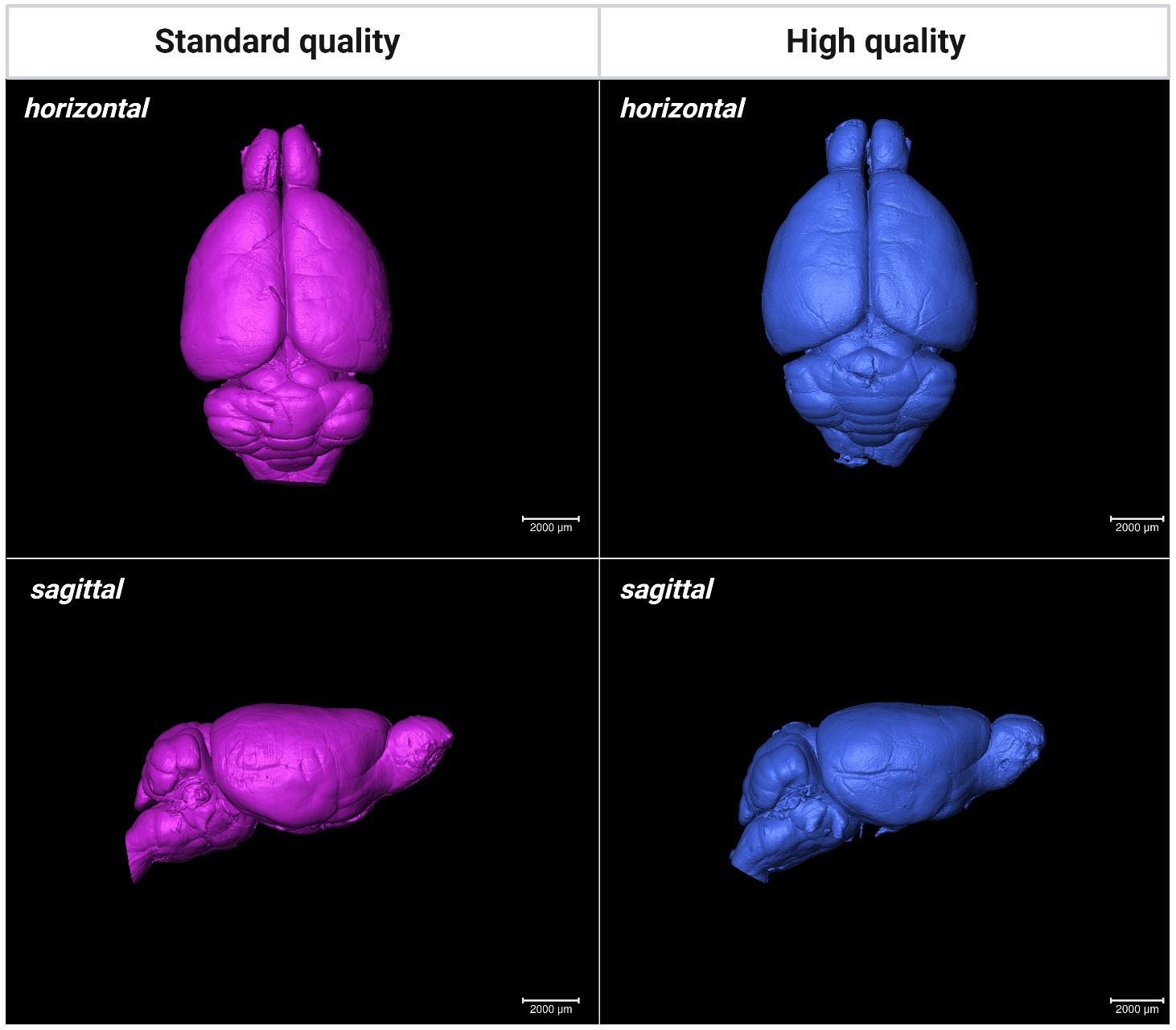


Supplementary Figure S5. Reconstructed 3D mouse brain surface rendering on sagittal and horizontal views. Sample from standard quality treatment is shown in pink and sample from high quality treatment is shown in blue.


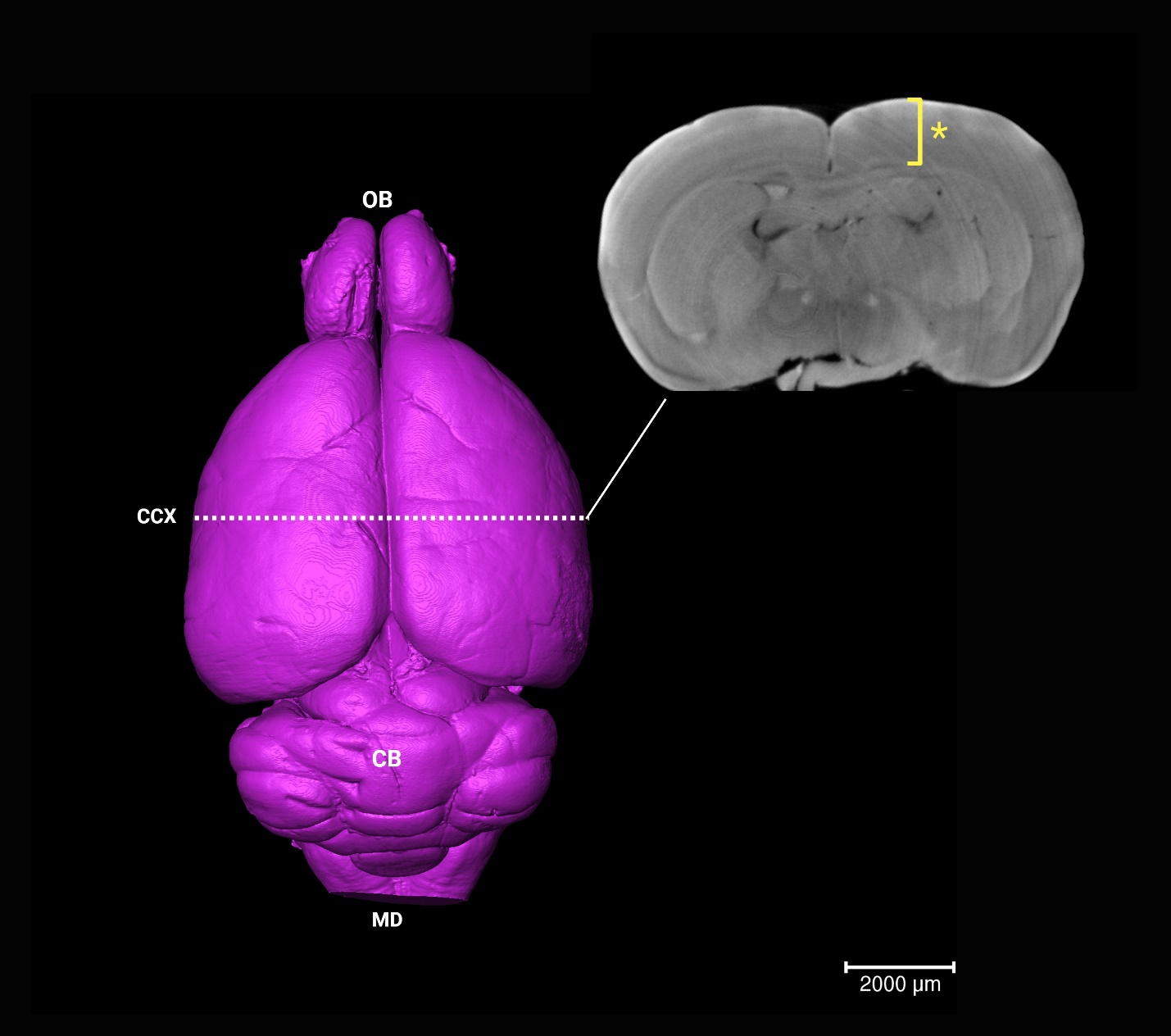


Supplementary Figure S6. Reconstructed 3D mouse brain surface rendering on horizontal view. Dotted line represents a cross-section of cerebral cortex. Asterix shows region used for cortex thickness measurements. (OB, olfactory bulbs; CCX, cerebral cortex; CB, cerebellum; MD, medulla).

Supplementary Table S2– Number of animals used in behavioural tests

| **Number of animals** | | | |
| --- | --- | --- | --- |
| Generation | SQ | HQ | Total |
| Open Field | | | |
| F3 | 36 | 41 | 77 |
| F4 | 50 | 51 | 101 |
| F5 | 50 | 50 | 100 |
| F5 cages | 30 | 29 | 59 |
| F6 cages | 45 | 48 | 93 |
| Novel Object | | | |
| F5 cages | 21 | 18 | 39 |
| Escape problem solving | | | |
| F3 | 30 | 21 | 51 |
| Food rewarded problem solving | | | |
| F5 cages | 21 | 18 | 39 |
| F6 cages | 7 | 8 | 15 |
| Reversal learning: | | | |
| Habituation | | | |
| F5 cages | 19 | 13 | 32 |
| F6 cages | 7 | 8 | 15 |
| Training | | | |
| F5 cages | 18 | 13 | 31 |
| F6 cages | 6 | 5 | 11 |
| Learning | | | |
| F5 cages | 15 | 10 | 25 |
| F6 cages | 4 | 4 | 8 |
| Reversal | | | |
| F5 cages | 15 | 10 | 25 |
| F6 cages | 4 | 3 | 7 |

**Summary tables of model outputs**

**Open Field estimates:**

Supplementary Table S3 Output of LMM for F3 mice on SQ diet from semi-natural environment for OF test

| **OF_distance** | | |  |
| --- | --- | --- | --- |
| *Predictors* | *Estimates* | *CI* | *p* |
| (Intercept) | 5716.99 | 4985.36 – 6448.61 | **<0.001** |
| sex [m] | -116.12 | -694.75 – 462.52 | 0.690 |
| trial | -1228.15 | -1676.81 – -779.48 | **<0.001** |
| **Random Effects** | | | |
| σ^2^ | 732410.75 | | |
| τ_00_ _ID_ | 361629.14 | | |

Supplementary Table S4 Output of LMM for F3 mice on HQ diet from semi-natural environment for OF test

| **OF_distance** | | |  |
| --- | --- | --- | --- |
| *Predictors* | *Estimates* | *CI* | *p* |
| (Intercept) | 1768.25 | 968.82 – 2567.68 | **<0.001** |
| sex [m] | -64.61 | -907.50 – 778.28 | 0.879 |
| trial | 263.08 | 10.38 – 515.79 | **0.042** |
| **Random Effects** | | | |
| σ^2^ | 272498.69 | | |
| τ_00_ _ID_ | 1497508.21 | | |

Supplementary Table 5 Output of LMM for F4 mice on SQ diet from semi-natural environment for OF test

| **OF_distance** | | |  |
| --- | --- | --- | --- |
| *Predictors* | *Estimates* | *CI* | *p* |
| (Intercept) | 3902.53 | 3189.92 – 4615.15 | **<0.001** |
| sex [m] | -168.20 | -609.69 – 273.30 | 0.449 |
| trial | -473.77 | -992.65 – 45.12 | 0.073 |
| **Random Effects** | | | |
| σ^2^ | 775534.17 | | |
| τ_00_ _ID_ | 0.00 | | |

Supplementary Table S6 Output of LMM for F4 mice on HQ diet from semi-natural environment for OF test

| **OF_distance** | | |  |
| --- | --- | --- | --- |
| *Predictors* | *Estimates* | *CI* | *p* |
| (Intercept) | 268.81 | -515.04 – 1052.66 | 0.495 |
| sex [m] | -520.68 | -1250.32 – 208.96 | 0.159 |
| trial | 2176.69 | 1663.36 – 2690.02 | **<0.001** |
| **Random Effects** | | | |
| σ^2^ | 553366.40 | | |
| τ_00_ _ID_ | 1220360.47 | | |

Supplementary Table S7 Output of LMM for F5 mice on SQ diet from semi-natural environment for OF test

|  | **OF_distance** | | |
| --- | --- | --- | --- |
| *Predictors* | *Estimates* | *CI* | *p* |
| (Intercept) | 4725.07 | 3916.82 – 5533.31 | **<0.001** |
| sex [m] | -621.67 | -1167.79 – -75.55 | **0.026** |
| trial | -737.48 | -1246.29 – -228.67 | **0.005** |
| **Random Effects** | | | |
| σ^2^ | 848092.16 | | |
| τ_00_ _ID_ | 261781.78 | | |

Supplementary Table S8 Output of LMM for F5 mice on HQ diet from semi-natural environment for OF test

|  | **OF_distance** | | |
| --- | --- | --- | --- |
| *Predictors* | *Estimates* | *CI* | *p* |
| (Intercept) | 4348.56 | 3714.79 – 4982.32 | **<0.001** |
| sex [m] | -510.07 | -987.25 – -32.89 | **0.037** |
| trial | -51.43 | -518.28 – 415.42 | 0.826 |
| **Random Effects** | | | |
| σ^2^ | 584659.16 | | |
| τ_00_ _ID_ | 174898.06 | | |

Supplementary Table S9 Output of LMM for F5* mice on SQ diet from cage housing for OF test

| **OF_distance** | | |  |
| --- | --- | --- | --- |
| *Predictors* | *Estimates* | *CI* | *p* |
| (Intercept) | 6044.44 | 5060.76 – 7028.12 | **<0.001** |
| sex [m] | 657.51 | -38.65 – 1353.68 | 0.064 |
| trial | -1150.11 | -1724.43 – -575.78 | **<0.001** |
| **Random Effects** | | | |
| σ^2^ | 1205774.27 | | |
| τ_00_ _ID_ | 288821.31 | | |

Supplementary Table S10 Output of LMM for F5* mice on HQ diet from cage housing for OF test

| **OF_distance** | | |  |
| --- | --- | --- | --- |
| *Predictors* | *Estimates* | *CI* | *p* |
| (Intercept) | 6864.28 | 5780.54 – 7948.01 | **<0.001** |
| sex [m] | -939.79 | -1718.28 – -161.29 | **0.019** |
| trial | -861.37 | -1511.60 – -211.14 | **0.011** |
| **Random Effects** | | | |
| σ^2^ | 1262434.90 | | |
| τ_00_ _ID_ | 338519.05 | | |

Supplementary Table S11 Output of LMM for F6* mice on SQ diet from cage housing for OF test

| **OF_distance** | | |  |
| --- | --- | --- | --- |
| *Predictors* | *Estimates* | *CI* | *p* |
| (Intercept) | 2817.61 | 2245.82 – 3389.41 | **<0.001** |
| sex [m] | 277.09 | -332.93 – 887.10 | 0.367 |
| trial | -427.84 | -702.84 – -152.85 | **0.003** |
| **Random Effects** | | | |
| σ^2^ | 190778.37 | | |
| τ_00_ _ID_ | 1058592.90 | | |

Supplementary Table S12 Output of LMM for F6* mice on HQ diet from cage housing for OF test

| **OF_distance** | | |  |
| --- | --- | --- | --- |
| *Predictors* | *Estimates* | *CI* | *p* |
| (Intercept) | 3087.07 | 1901.36 – 4272.78 | **<0.001** |
| sex [m] | 566.22 | -603.50 – 1735.94 | 0.338 |
| trial | 15.28 | -689.65 – 720.21 | 0.966 |
| **Random Effects** | | | |
| σ^2^ | 1674818.24 | | |
| τ_00_ _ID_ | 2861633.04 | | |

**Novel object estimates:**

Supplementary Table S13 Output of LMM for F5* mice on both diet from cage housing for Novel Object

|  | **time** | | |
| --- | --- | --- | --- |
| *Predictors* | *Estimates* | *CI* | *p* |
| (Intercept) | 52.48 | 25.13 – 79.84 | **<0.001** |
| food | 1.15 | -12.80 – 15.11 | 0.870 |
| trial | -7.29 | -18.53 – 3.94 | 0.200 |
| **Random Effects** | | | |
| σ^2^ | 619.60 | | |
| τ_00_ _ID_ | 165.52 | | |
| ICC | 0.21 | | |
| N _ID_ | 39 | | |
| Observations | 78 | | |
| Marginal R^2^ / Conditional R^2^ | 0.017 / 0.224 | | |

Supplementary Table S14 Output of LMM for F5* mice on SQ diet from cage housing for Novel Object

| **latency** | | |  |
| --- | --- | --- | --- |
| *Predictors* | *Estimates* | *CI* | *p* |
| (Intercept) | 104.81 | -351.24 – 560.86 | 0.644 |
| trial | 92.48 | -195.96 – 380.91 | 0.520 |
| **Random Effects** | | | |
| σ^2^ | 213153.55 | | |
| τ_00_ _ID_ | 0.00 | | |
| N _ID_ | 21 | | |
| Observations | 42 | | |
| Marginal R^2^ / Conditional R^2^ |  |  |  |

Supplementary Table S15 Output of LMM for F5* mice on HQ diet from cage housing for Novel Object

|  | **latency** | | |
| --- | --- | --- | --- |
| *Predictors* | *Estimates* | *CI* | *p* |
| (Intercept) | 293.78 | 135.74 – 451.82 | **0.001** |
| trial | -105.39 | -190.58 – -20.20 | **0.017** |
| **Random Effects** | | | |
| σ^2^ | 15741.01 | | |
| τ_00_ _ID_ | 29648.53 | | |
| ICC | 0.65 | | |
| N _ID_ | 18 | | |

Supplementary Table S16 Output of LMM for F5* mice on both diet from cage housing for Novel Object

|  | **time** | | |  |
| --- | --- | --- | --- | --- |
| *Predictors* | *Estimates* | *CI* | *p* |  |
| (Intercept) | 52.48 | 25.13 – 79.84 | **<0.001** |  |
| food | 1.15 | -12.80 – 15.11 | 0.870 |  |
| trial | -7.29 | -18.53 – 3.94 | 0.200 |  |
| **Random Effects** | | | |  |
| σ^2^ | 619.60 | | |  |
| τ_00_ _ID_ | 165.52 | | |  |
| ICC | 0.21 | | |  |
| N _ID_ | 39 | | |  |
| Observations | 78 | | |  |
| Marginal R^2^ / Conditional R^2^ | 0.017 / 0.224 | | |  |

Supplementary Table S17 Output of LMM for F5* mice on SQ diet from cage housing for Novel Object

|  |  |  |  |
| --- | --- | --- | --- |
| **time** | | |  |
| *Predictors* | *Estimates* | *CI* | *p* |
| (Intercept) | 52.13 | 27.74 – 76.51 | **<0.001** |
| trial | -6.28 | -21.34 – 8.77 | 0.404 |
| **Random Effects** | | | |
| σ^2^ | 580.88 | | |
| τ_00_ _ID_ | 143.41 | | |
| ICC | 0.20 | | |
| N _ID_ |  |  |  |

Supplementary Table S18 Output of LMM for F5* mice on HQ diet from cage housing for Novel Object

| **time** | | |  |
| --- | --- | --- | --- |
| *Predictors* | *Estimates* | *CI* | *p* |
| (Intercept) | 56.55 | 27.45 – 85.66 | **<0.001** |
| trial | -8.47 | -26.43 – 9.50 | 0.344 |
| **Random Effects** | | | |
| σ^2^ | 700.24 | | |
| τ_00_ _ID_ | 173.99 | | |
| ICC | 0.20 | | |
| N _ID_ | 18 | | |

Supplementary Table S19 Output of LMM for F5* mice on both diet from cage housing for Novel Object

|  | **number** | | |
| --- | --- | --- | --- |
| *Predictors* | *Incidence Rate Ratios* | *CI* | *p* |
| (Intercept) | 4.46 | 2.51 – 7.95 | **<0.001** |
| food | 1.33 | 0.94 – 1.88 | 0.112 |
| trial | 1.27 | 1.12 – 1.45 | **<0.001** |
| **Random Effects** | | | |
| σ^2^ | 0.10 | | |
| τ_00_ _ID_ | 0.25 | | |
| ICC | 0.72 | | |
| N _ID_ | 39 | | |
| Observations | 78 | | |
| Marginal R^2^ / Conditional R^2^ | 0.090 / 0.746 | | |

Supplementary Table S20 Output of LMM for F5* mice on SQ diet from cage housing for Novel Object

|  | **number** | | |
| --- | --- | --- | --- |
| *Predictors* | *Incidence Rate Ratios* | *CI* | *p* |
| (Intercept) | 12.73 | 9.14 – 17.72 | **<0.001** |
| trial | 0.78 | 0.64 – 0.95 | **0.014** |
| **Random Effects** | | | |
| σ^2^ | 0.11 | | |
| τ_00_ _ID_ | 0.09 | | |
| ICC | 0.44 | | |
| N _ID_ | 21 | | |
| Observations | 42 | | |
| Marginal R^2^ / Conditional R^2^ | 0.077 / 0.485 | | |

Supplementary Table S21 Output of LMM for F5* mice on HQ diet from cage housing for Novel Object

|  | **number** | | |
| --- | --- | --- | --- |
| *Predictors* | *Incidence Rate Ratios* | *CI* | *p* |
| (Intercept) | 4.08 | 2.64 – 6.32 | **<0.001** |
| trial | 1.89 | 1.57 – 2.26 | **<0.001** |
| **Random Effects** | | | |
| σ^2^ | 0.09 | | |
| τ_00_ _ID_ | 0.42 | | |
| ICC | 0.83 | | |
| N _ID_ | 18 | | |
| Observations | 36 | | |
| Marginal R^2^ / Conditional R^2^ | 0.170 / 0.858 | | |
